# Glycogen synthase kinase 3β: the nexus of chemoresistance, invasive capacity, and cancer stemness in pancreatic cancer

**DOI:** 10.20517/cdr.2023.84

**Published:** 2024-01-31

**Authors:** Masahiro Uehara, Takahiro Domoto, Satoshi Takenaka, Osamu Takeuchi, Takeo Shimasaki, Tomoharu Miyashita, Toshinari Minamoto

**Affiliations:** ^1^Division of Translational and Clinical Oncology, Cancer Research Institute, Kanazawa University, Kanazawa 920-0934, Japan.; **^2^** Department of Hepato-Biliary-Pancreatic Surgery and Transplantation, Graduate School of Medical Sciences, Kanazawa University, Kanazawa 920-8641, Japan.; ^3^Department of Surgery, Toyama City Hospital, Toyama 939-8511, Japan.; ^4^Biomedical Laboratory, Department of Research, Kitasato University Kitasato Institute Hospital, Tokyo 108-8642, Japan.; ^5^Medical Research Institute, Kanazawa Medical University, Uchinada 920-0293, Japan.; ^#^Authors contributed equally.

**Keywords:** Pancreatic cancer, chemoresistance, tumor invasion, cancer stemness, glycogen synthase kinase 3β

## Abstract

The treatment of pancreatic cancer remains a significant clinical challenge due to the limited number of patients eligible for curative (R0) surgery, failures in the clinical development of targeted and immune therapies, and the pervasive acquisition of chemotherapeutic resistance. Refractory pancreatic cancer is typified by high invasiveness and resistance to therapy, with both attributes related to tumor cell stemness. These malignant characteristics mutually enhance each other, leading to rapid cancer progression. Over the past two decades, numerous studies have produced evidence of the pivotal role of glycogen synthase kinase (GSK)3β in the progression of over 25 different cancer types, including pancreatic cancer. In this review, we synthesize the current knowledge on the pathological roles of aberrant GSK3β in supporting tumor cell proliferation and invasion, as well as its contribution to gemcitabine resistance in pancreatic cancer. Importantly, we discuss the central role of GSK3β as a molecular hub that mechanistically connects chemoresistance, tumor cell invasion, and stemness in pancreatic cancer. We also discuss the involvement of GSK3β in the formation of desmoplastic tumor stroma and in promoting anti-cancer immune evasion, both of which constitute major obstacles to successful cancer treatment. Overall, GSK3β has characteristics of a promising therapeutic target to overcome chemoresistance in pancreatic cancer.

## INTRODUCTION

Approximately one-third of patients diagnosed with pancreatic ductal adenocarcinoma (PDAC) present with locally unresectable tumors, while nearly half exhibit distant metastatic tumors. Consequently, only 10%-15% of stage I or II patients can undergo resectable (R0) surgery, a significant proportion of whom eventually suffer local disease recurrence and/or distant metastasis post-surgery^[1,2]^. First-line chemotherapeutic regimens for palliative treatment of locally advanced pancreatic cancer and metastatic cases include FOLFIRINOX [a combination of folate, 5-fluorouracil (FU), irinotecan, and oxaliplatin]^[3]^, and a combination of nanoparticle albumin-bound (nab)-paclitaxel and gemcitabine^[4]^. The second-line regimen is FOLFIRI^[5]^. These multi-agent chemotherapy regimens offer only a modestly improved efficacy compared to gemcitabine monotherapy, and are appropriate only for a small proportion of pancreatic cancer patients with a good performance status (PS 0 or 1)^[3-6]^. Patients with a PS of 2 or higher, who represent most pancreatic cancer cases, undergo gemcitabine monotherapy^[7,8]^, with many quickly developing resistance to the drug^[9-11]^. Unlike lung and colorectal cancer, neither preclinical studies nor clinical trials have yet to demonstrate significant efficacy against pancreatic cancer of rationally targeted agents, precision medicines based on the identification of actionable proto-oncoproteins, or immune checkpoint blockade^[12-20]^. Given these circumstances, the development of biology-based strategies against chemoresistance, specifically the resistance to gemcitabine by pancreatic cancer, is a pressing need in pancreatic cancer research^[21]^.

## MECHANISMS OF GEMCITABINE RESISTANCE IN PANCREATIC CANCER

Many reviews^[9-11,22-25]^ have thoroughly examined the mechanisms of action of, and resistance to, gemcitabine in pancreatic cancer cells, detailing the steps of intra- and extracellular drug transportation^[26-29]^, metabolic activation of gemcitabine^[30-35]^, molecular pathways that combat drug-induced apoptosis^[36-41]^, pro-oncogenic pathways that enable tumor cells to survive cytotoxic effects^[36,42-51]^, and epithelial-mesenchymal transition (EMT), a pro-invasive tumor property^[52,53]^. These reviews^[10,11,22-25]^ also discuss the role of specific micro-RNAs in altering the expression of molecules linked to gemcitabine resistance. Besides the resistance mechanisms emerging in pancreatic cancer cells, comprehensive reviews have discussed the many mechanisms for chemoresistance, including resistance to gemcitabine, targeted agents, and immune checkpoint blockades, brought on by tumor cell interactions with the tumor microenvironment (TME)^[54-63]^ [Supplementary Table 1].

Efforts have been made to address resistance mechanisms that interfere with gemcitabine uptake and activation, with several compounds developed to enhance nucleoside transporter (NP) expression or bypass NPs and deoxycytidine kinase (dCK) through different mechanisms or chemical modifications of gemcitabine^[9,11,64]^. Several experimental studies have sought to reverse chemoresistance by activating multiple cell death pathways^[65]^, deconstructing the desmoplastic stroma, and targeting immunosuppressive pathways within the hostile TME in pancreatic cancer^[66-74]^. Despite a decade of extensive research efforts, there has been limited therapeutic advancement in preclinical or clinical settings. This reaffirms the urgent need to identify mechanism-based strategies and therapeutic targets to overcome gemcitabine resistance in pancreatic cancer research.

## MUTUAL DEPENDENCY OF CHEMORESISTANCE, TUMOR CELL INVASION, AND STEMNESS IN PANCREATIC CANCER

One of the fundamental challenges in addressing cancer drug resistance stems from the complex biological properties that tumor cells inherently possess (e.g., intrinsic resistance) or acquire during exposure to therapeutic agents (acquired resistance)^[75]^. Recent comprehensive reviews have detailed these properties, which include altered pharmacological reactions of tumor cells to drugs and their intermediate metabolites, clonal evolution of tumor cells to generate resistant clones, the latency and plasticity of cancer stem cells or tumor cells’ resilience to acquiring stemness phenotype, genetic and biological heterogeneity of tumor cells, activation of pro-survival pathways, impairment in cell death pathways, and adaptation to therapeutic pressures^[76,77]^. The aforementioned properties synergistically lead to increased resistance to therapy in various cancer types^[77]^.

**Figure 1:**
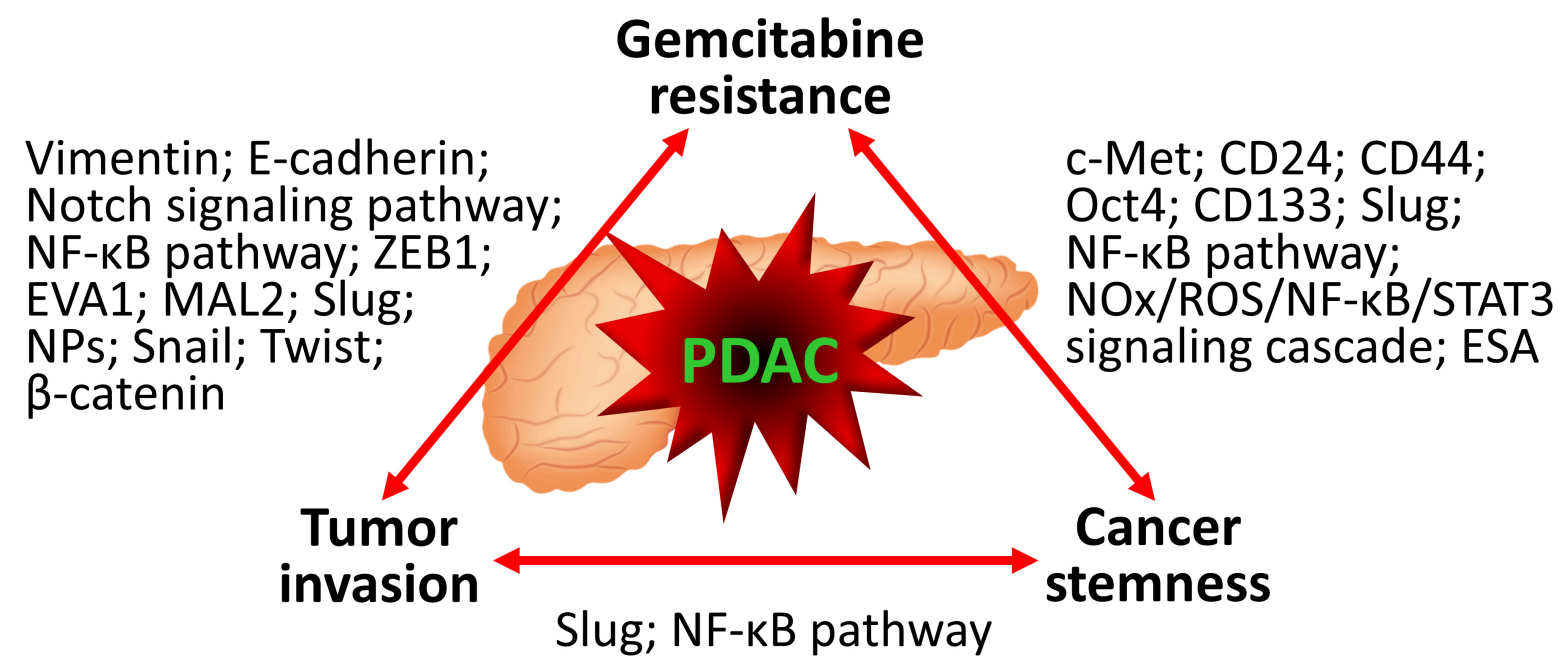
The mechanistic interconnection of chemoresistance, tumor invasion, and cancer stemness presents in chemoresistant pancreatic cancer. Mechanisms responsible for the respective connections are described in Table 1. NF-κB: Nuclear factor-κB; ZEB1: zinc-finger-enhancer binding protein 1; EVA1: epithelial V-like antigen 1; MAL2: myelin and lymphocyte protein 2; NPs: nucleoside transporters; CD: cluster of differentiation; Oct4: octamer-binding transcription factor 4; NOx: nitrogen oxides; ROS: reactive oxygen species; STAT3: signal transducer and activator of transcription 3; ESA: epithelial-specific antigen; PDAC: pancreatic ductal adenocarcinoma.

The concept of cancer stem cells (CSCs) has been proposed based on the observation that the uncontrolled proliferation of cancer is driven by a biologically distinct subset of tumor cells that are present in a relatively small proportion within the whole tumor^[78,79]^. CSCs are characterized by their ability to self-renew, sustain tumor propagation, express specific cell surface markers, and use multidrug efflux pumps. The clinical implication of CSCs lies in their potential to drive tumor regrowth after seemingly successful treatment with chemotherapeutics, radiation, or targeted agents because of their inherent resistance to therapy^[80-83]^. This has positioned CSCs as a critical therapeutic target, albeit one that is currently elusive due to their complex biological nature in intractable cancer types, including pancreatic cancer^[84-90]^. Several studies have shown that cancer therapy spares not only CSCs but also some residual cancer cells that acquire a CSC-like phenotype without mutation-based clonal selection, thus becoming resistant to therapy. This phenotypic switch is often associated with the activation of pro-oncogenic pathways such as Wnt- and Notch-mediated pathways^[91]^. Consistently, gemcitabine treatment can induce a shift towards a cancer stemness phenotype, primarily in gemcitabine-naïve pancreatic cancer cells^[53,92-95]^ [Table 1, Figure 1].

**Table 1:** Tumor stemness and pro-invasive properties of PDAC cells surviving the insult from continuous or repeated exposure to gemcitabine

	**Mechanisms for gemcitabine-induced phenotypes**	**Ref.**
**Cancer stemness** **phenotype**	Acquisition of resistance to gemcitabine imparts stemness phenotype to the PDAC cells via the phosphorylation-mediated activation of c-Met receptor protein tyrosine kinase and the increased expression of CSC markers CD24 and CD44	[53]
	PDAC cells surviving gemcitabine treatment express the CSC markers: CD44, CD24, Oct4, and CD133, and an EMT marker slug	[92]
	Gemcitabine-resistant PDAC cells acquire cancer stemness and EMT phenotypes mediated by the NF-κB pathway	[93]
	Gemcitabine treatment promotes chemoresistance and cancer stemness through the NOx/ROS/NF-κB/STAT3 signaling cascade	[94]
	Gemcitabine-resistant PDAC cells show the activation of c-Met receptor protein tyrosine kinase and the increased expression of CSC markers CD24, CD44, and ESA	[95]
**Pro-invasive phenotype**	Gemcitabine-resistant PDAC cells show spindle shape with pseudopodia, increased expression of vimentin, and decreased expression of E-cadherin	[53]
	Gemcitabine-resistant PDAC cells acquire EMT phenotype by activation of the Notch signaling pathway	[49]
	Gemcitabine-resistant PDAC cells show the gene expression profile responsible for EMT phenotype	[115]
	PDAC cells surviving gemcitabine treatment express the CSC markers: CD44, CD24, Oct4, and CD133, and an EMT marker slug	[92]
	Gemcitabine-resistant PDAC cells acquire cancer stemness and EMT phenotypes mediated by the NF-κB pathway	[93]
	Gemcitabine-resistant PDAC cells showed increased expression of ZEB1	[52]
	Suppression of EMT leads to an increase in PDAC cell proliferation with enhanced expression of NPs in tumors, contributing to enhanced sensitivity to gemcitabine and increased survival of mouse models of PDAC with deletion of snail or twist	[11]
	Gemcitabine-resistant PDAC cells show spindle shape with pseudopodia, migratory and invasive capacity, increased vimentin and decreased E-cadherin expression, and nuclear localization of β-catenin	[95]

PDAC: Pancreatic ductal adenocarcinoma; CSC: cancer stem cell; CD: cluster of differentiation; Oct4: octamer-binding transcription factor 4; EMT: epithelial-mesenchymal transition; NF-κB: nuclear factor-κB; NOx: nitrogen oxides; ROS: reactive oxygen species; STAT3: signal transducer and activator of transcription 3; ESA: epithelial-specific antigen; ZEB1: zinc-finger-enhancer binding protein 1; NPs: nucleoside transporters.

Highly invasive capacity and therapy resistance are the defining biological and clinical characteristics of pancreatic cancer, often resulting in treatment failure and poor patient outcomes^[1,2]^. Although not sufficient on its own, tumor invasion serves as an initial step in the complex process leading to cancer progression and metastasis^[96]^. EMT, which modifies the morphological and functional behaviors of cancer cells to resemble mesenchymal cell types, is a key prerequisite for the invasion seen in many cancer types^[97,98]^, including pancreatic cancer^[99]^. A growing body of research has demonstrated the causal link between chemotherapeutic stimuli and EMT in various cancer types, such as colon^[100,101]^, ovarian^[102]^, breast^[103-107]^, and skin squamous cell carcinoma^[108]^. These studies have given rise to a concept that chemoresistance and the tumor invasion-metastasis cascade are interrelated processes that accelerate cancer progression^[109-112]^. In line with this concept and the paradox of treatment-induced metastasis, suggesting that nearly all cancer treatments can inadvertently trigger and facilitate metastatic spread^[113,114]^, several studies have reported an association of resistance to gemcitabine with EMT and invasion in pancreatic cancer^[49,52,53,92,93,95,115,116]^ [Table 1, Figure 1].

**Figure 2:**
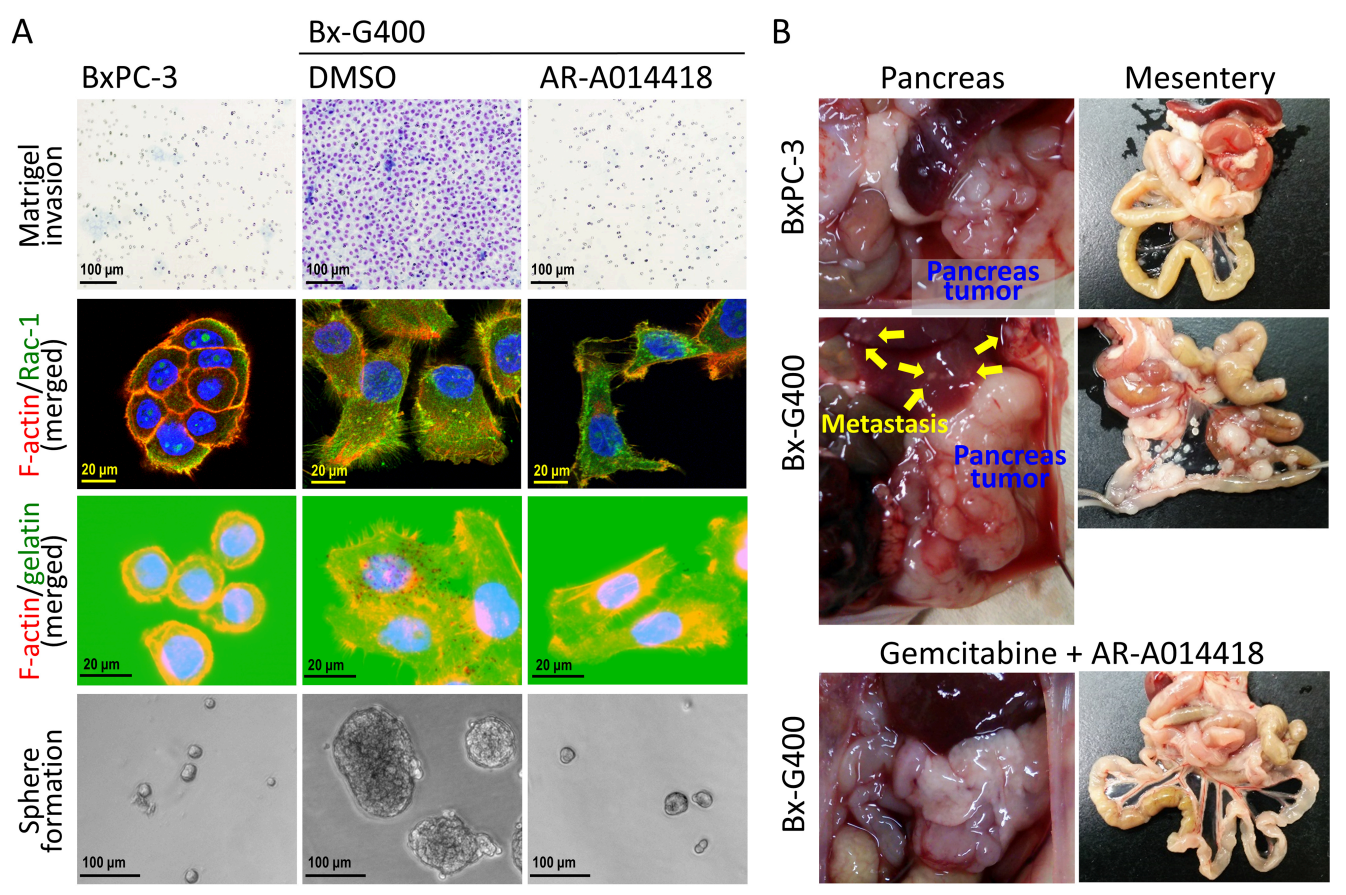
(A) Representative findings of matrigel invasion (upper panels), formation of lamellipodia and invadopodia [middle six panels: nuclei were counterstained with DAPI (blue fluorescence), and sphere formation (lower panels) of BxPC-3 cells and BxPC-3 cell-derived gemcitabine-resistant Bx-G400 cells that were treated with DMSO (diluent of the inhibitor) and GSK3β inhibitor, AR-A014418; (B) Laparotomy findings of the mice with intrapancreatic transplantation of BxPC-3 and Bx-G400 cells, respectively. The bottom panels show the mice treated with gemcitabine and AR-A014418 in combination for 8 weeks after transplantation. DAPI: 4’,6-diamidino-2-phenylindole; DMSO: dimethyl sulfoxide; GSK3β: glycogen synthase kinase 3β.

Regardless of therapy resistance, mounting evidence has shown a strong association between the pro-invasive phenotype (represented by EMT) and CSCs in many cancer types^[117-120]^, including pancreatic cancer^[121]^, because of shared distinct biological mechanisms. Consistent with this connection, many of the studies referenced in Table 1 have reported a simultaneous association of resistance to gemcitabine with both pro-invasive and cancer stemness phenotypes^[52,53,92,93,95]^. This highlights a three-way (or triangular) interaction among chemoresistance, tumor invasion, and cancer stemness in pancreatic cancer [Figure 1], as previously suggested^[122,123]^. We have established gemcitabine-sensitive pancreatic cancer BxPC-3 derivative cell clones that gained stepwise resistance to gemcitabine (BxG30, BxG140, and BxG400 in increasing order of resistance)^[124]^. We have recently observed an increased invasive capacity with the formation of characteristic cellular surface microstructures (lamellipodia^[125]^ and invadopodia^[126]^), and sphere-forming ability in the resistant cell clones compared to their parental BxPC-3 cells [Figure 2A]. Moreover, we have observed severe local tumor invasion and metastasis to the liver and peritoneum in mice orthotopically transplanted with the most resistant BxG400 cells [Figure 2B], which models the refractory pancreatic cancer patients developing resistance to gemcitabine. Our preliminary observations reaffirm the interdependency between these malignant properties in pancreatic cancer cells acquiring resistance to gemcitabine.

## OUTLINE OF GLYCOGEN SYNTHASE KINASE 3β BIOLOGY AND ITS INVOLVEMENT IN DISEASES

Glycogen synthase kinase (GSK)3β is an isoform of the GSK3 family of serine (S)/threonine (T) protein kinases. GSK3β is involved in a multitude of biological processes and pathways in the complex molecular networks of cells, where it interacts with nearly 100 or more structural and functional proteins via phosphorylation^[127,128]^. The enzymatic activity of GSK3β is regulated by the differential phosphorylation of its S9 (inactive form) and tyrosine (Y)216 (active form) residues. Even though GSK3β is typically active in cells, inhibitory regulation of its activity primarily benefits normal cells in maintaining their vital activity and homeostasis in response to various extracellular and intracellular stimuli^[127-129]^. Due to the redundancy in cellular expression and functions between GSK3β and its isoform GSK3α, the pathological roles of GSK3β have garnered increased attention. Aberrant activity and expression of GSK3β, as well as defects in its inhibitory regulation, contribute to the pathogenesis and progression of common diseases, including type 2 diabetes mellitus, neurodegenerative diseases (e.g., Alzheimer’s disease), inflammatory and immunological diseases, and cancer^[130,131]^. Given its counteracting functions in normal cells and diseases, GSK3β is considered a potential therapeutic target in major health disorders, thereby driving the identification and development of pharmacological GSK3β inhibitors^[132-136]^.

The biochemistry, biology, and functions of GSK3 family kinases (GSK3α and GSK3β) in normal cells, as well as their involvement in a wide range of common diseases, have increasingly attracted scientific attention in biomedical and pharmacological fields. This topic has been extensively reviewed in previous literature^[127-136]^, and is therefore only briefly outlined here.

## TUMOR-PROMOTING ROLES OF GSK3β IN PANCREATIC CANCER

**Figure 3:**
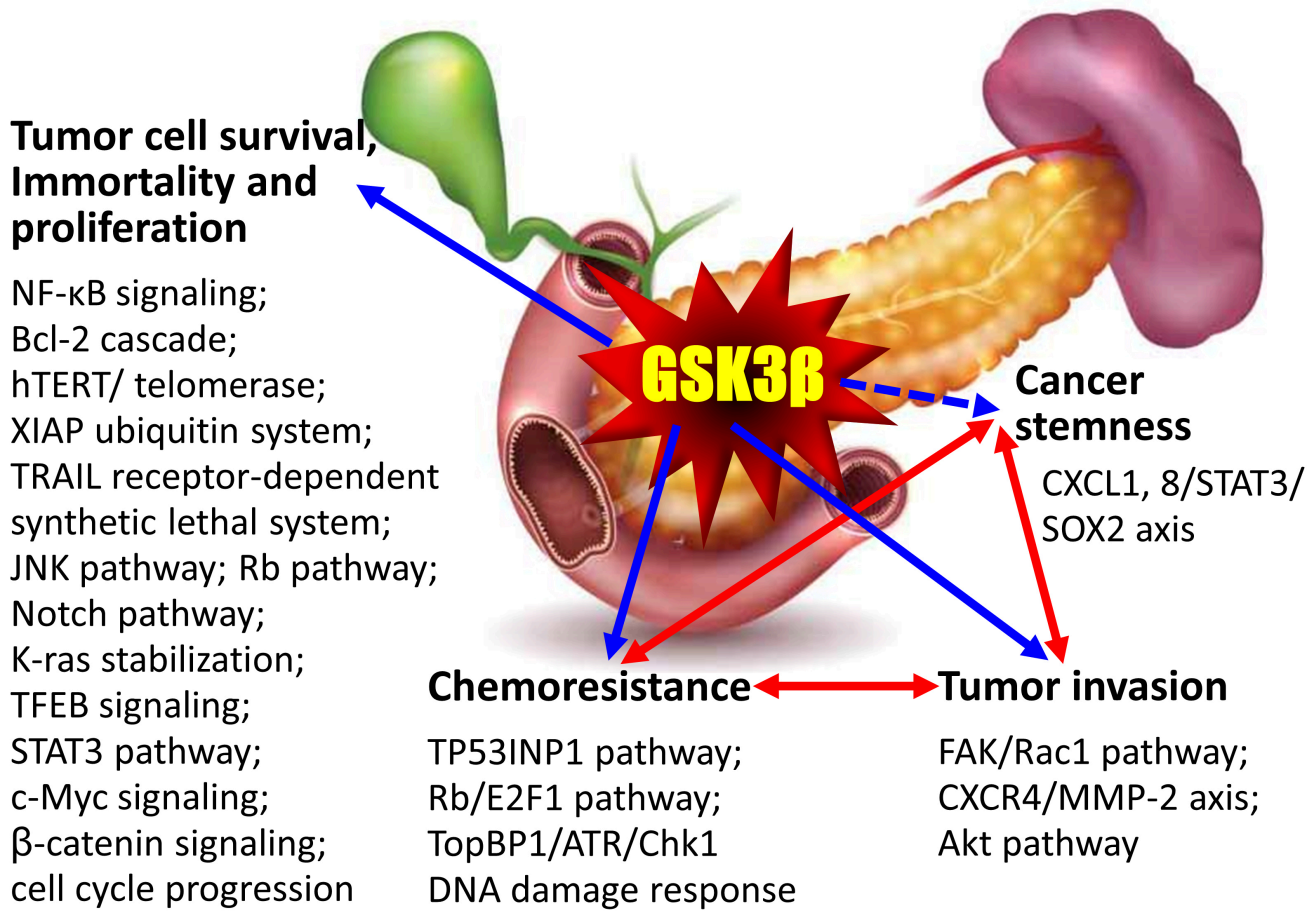
Tumor-promoting properties of GSK3β and underlying mechanisms reported in pancreatic cancer^[156-182]^. Dotted arrow: preliminary findings (by Domoto *et al.*). The mechanisms responsible for the respective interconnections indicated by red bidirectional arrows are shown in Table 1 and Figure 1. GSK3β: Glycogen synthase kinase 3β; NF-κB: nuclear factor κB; Bcl-2: B-cell/chronic lymphocytic leukemia lymphoma 2; hTERT: human telomerase reverse transcriptase; XIAP: X-linked inhibitor of apoptosis protein; TRAIL: tumor necrosis factor-related apoptosis-inducing ligand; JNK: c-Jun N-terminal kinase; TFEB: transcription factor EB; STAT3: signal transducer and activator of transcription 3; TP53INP1: tumor protein 53-inducible nuclear protein 1; E2F1: E2 transcription factor 1; TopBP1: topoisomerase II binding protein 1; ATR: ataxia telangiectasia and Rad3-related; Chk1: checkpoint kinase 1; FAK: focal adhesion kinase; CXCR4: C-X-C chemokine receptor type 4; MMP: matrix metalloproteinase; CXCL: C-X-C chemokine ligand; SOX2: sex-determining region Y-box transcription factor 2.

Contrary to its pathological roles in diseases other than cancer, activated GSK3β counters pro-oncogenic pathways such as those mediated by Wnt/β-catenin, Hedgehog, and Notch signaling and transcription factors (e.g., snail) that induce EMT in normal cells. Given its functions in non-transformed cells, activation of GSK3β has long been hypothesized to suppress the development and progression of cancer^[137-139]^, thereby posing a challenge to pharmaceutical industries and clinical oncologists aiming to develop and apply GSK3β inhibitors for cancer treatment. However, no direct evidence supports the tumor-suppressor role of this kinase, nor the effect of GSK3β inhibition on promoting cancer development and progression. Contrary to this hypothesis, a significant amount of research conducted by our group and others over the last two decades has provided solid evidence demonstrating the tumor-promoting roles of active GSK3β as well as therapeutic effects related to its inhibition in more than 25 different cancer types (reviewed in^[140-146]^), including pancreatic cancer (reviewed in^[147-152]^). As a result, GSK3β has emerged as a potential therapeutic target in cancer, encouraging the development of GSK3β inhibitors for cancer treatment^[153-155]^. Similar to other cancer types, accumulating evidence for pancreatic cancer^[156-182]^ shows that deregulated GSK3β supports tumor cell survival, immortality, and proliferation by mediating distinct pathways. As discussed below, GSK3β also facilitates the invasion of tumor cells and makes them unresponsive or resistant to chemotherapy and ionizing radiation [Figure 3].

## GSK3β AS A MOLECULAR HUB IN MECHANISTICALLY WIRING THE CHEMORESISTANCE, TUMOR INVASION AND CANCER STEMNESS

As depicted in Figure 3, most studies on pancreatic cancer have demonstrated that the deregulated GSK3β sustains tumor cell survival, immortalization, and proliferation - common and fundamental features that engender therapy resistance in nearly all cancer types. This is achieved by enhancing cell immortality and several pro-oncogenic pathways [e.g., Nuclear factor-κB (NF-κB), Notch, K-ras, c-Myc], and by abrogating distinct tumor suppressor pathways (e.g., Rb) [Figure 3]. Our group and others have previously reported that GSK3β contributes to the unresponsiveness of pancreatic cancer cells to gemcitabine by impairing the DNA damage response mediated by tumor protein 53-inducible nuclear protein 1 (TP53INP1) and topoisomerase IIβ binding protein 1 (TopBP1)/ataxia telangiectasia and Rad3-related (ATR)/checkpoint kinase 1 (Chk1)^[165,169,178]^. We have also shown that GSK3β plays a role in the acquisition of resistance to gemcitabine in resistant pancreatic cancer cell clones derived from BxPC-3^[124]^ [Figure 2], via impairing the functional interaction between the Rb tumor suppressor protein and pro-oncogenic E2 transcription factor (E2F)1^[179]^. Our previous studies on gemcitabine-unresponsive pancreatic cancer and temozolomide-resistant glioblastoma cells have indicated that GSK3β enhances tumor cell migration and invasion via the focal adhesion kinase (FAK), Rac1, and c-Jun N-terminal kinase (JNK)-mediated pathway^[169,183]^. It is conceivable that the respective mechanisms responsible for chemoresistance and tumor invasion share GSK3β as a common trigger for both malignant properties in gemcitabine-resistant pancreatic cancer.

As we previously reviewed^[146]^, a series of studies have shown that GSK3β underpins the pivotal mechanisms for sustaining CSCs and the acquisition of cancer stemness phenotype in various cancer types, including colorectal cancer, prostate cancer, head and neck squamous cell carcinoma, glioblastoma, and leukemia. We have previously shown that kenpaullone, an adenosine triphosphate (ATP)-competitive GSK3β inhibitor, reversed the temozolomide resistance of glioblastoma patient-derived tumor stem cells^[184]^. In a recent preliminary study, we found that GSK3β inhibition counteracts tumor invasion and distant metastasis [Figure 2B] as well as the sphere formation of BxG400 cells, which acquire the highest resistance to gemcitabine established from the gemcitabine-sensitive pancreatic cancer BxPC-3 cells [Figure 2A]. These effects were associated with the suppression of C-X-C chemokine receptor type 4 (CXCR4)-mediated matrix metalloproteinase (MMP)-2 activation, and CXC ligand (CXCL) 1 and 8-induced activation of the signal transducer and activator of transcription (STAT)3/SRY-box transcription factor (SOX)2 axis (Figure 3; preliminary observations by Domoto *et al.*). Collectively, GSK3β potentially functions as a molecular hub that wires chemoresistance, tumor invasion, and cancer stemness phenotype, thereby aggravating pancreatic cancer towards the incurable/devastating disease stage.

## PRESUMPTIVE INVOLVEMENT OF GSK3β IN THE DESMOPLASTIC TUMOR STROMA AND THE PERMISSIVE ANTI-CANCER IMMUNE ENVIRONMENT

Desmoplastic tumor stroma and permissive (or tolerant) immunity against cancer make up the hostile TME^[60-62]^ that is recognized as a formidable obstacle for palliative treatment with chemotherapeutics, radiation, and targeted agents in pancreatic cancer^[70-73]^. Here, we briefly discuss the prospective involvement of GSK3β in the pancreatic cancer TME.

The primary cellular components of pancreatic cancer TME include pancreatic stellate cells (PSCs)^[185]^ and cancer-associated fibroblasts (CAFs)^[60,186,187]^. Both stromal cell types in pancreatic cancer support tumor cells and produce dense fibrous stroma that forms a physical barrier for drug delivery, and their interaction with tumor cells mechanistically contributes to chemoresistance. PSCs, a minor and quiescent cellular population in healthy pancreas stroma, are activated by extracellular stimulants including tumor necrosis factor (TNF)α, transforming growth factor (TGF)-β, and interleukin (IL)-1, IL-2, and IL-10 in the TME. Activated PSCs enable cancer cells to resist gemcitabine via the Notch pathway-mediated Hes1 overexpression^[188]^, exclusive expression of periostin in PSCs^[189]^, and the paracrine stromal cell-derived factor (SDF)-1α-mediated activation of FAK/Akt and extracellular signal-regulated kinase (ERK)1/2, with subsequent activation of the IL-6/Janus kinase (JAK)/STAT3 pathway in the tumor cells in an autocrine manner^[190]^. The cellular origin of CAFs includes bone marrow-derived mesenchymal stem cells (MSCs), PSCs, and preexisting dormant fibroblasts. CAFs are reportedly activated by sonic hedgehog (SHH), TGF-β, TNFα, and IL-1, IL-6, and IL-10. CAFs have been shown to support tumor cells through exosomal transfer and the paracrine signaling mediated by NF-κB and cytokines such as IL-6, thereby activating the downstream JAK/STAT3, mechanistic target of rapamycin (mTOR), and SHH pathways^[184,186]^. A previous study reported that CAFs with activation of the mTOR/4E-binding protein (BP)1 pathway and resultant secretion of IL-6 induced resistance to gemcitabine in pancreatic cancer cells^[191]^. While a previous study demonstrated that the GSK3β inhibitor BIO maintains proliferation and the undifferentiated state of immortalized pancreatic MSCs that share characteristics of bone marrow-derived MSCs and PSCs^[192]^, no studies have directly shown the role of GSK3β in PSCs or CAFs. As discussed above, the reported mechanisms by which PSCs and CAFs interact with and induce chemoresistance in pancreatic cancer cells involve the Notch, mTOR, and IL-6/JAK/STAT3 pathways. Previous reviews have described the roles of GSK3β in mediating Notch and mTOR signaling in different cancer types, including pancreatic cancer^[193,194]^. Previous studies have shown that GSK3β functions in the phosphorylation-dependent activation of STAT3^[195,196]^ and that inhibition of GSK3β attenuates the progression of gastric and esophageal cancers by suppressing STAT3 activity^[197,198]^. Therefore, elucidating the conceivable contribution of GSK3β to the interaction of PSCs and CAFs with tumor cells may provide a new strategy for targeting the tumor-promoting stroma, thereby combatting chemoresistance in pancreatic cancer.

The permissive immune environment in pancreatic cancer is complex, primarily resulting from the failure in innate immunity exerted by natural killer (NK) T-cells and the suppression of adaptive immunity by the immune checkpoint machinery between CD8+ T-cells and tumor cells, mediated by the programmed death (PD)-1/PD ligand (PD-L)1 axis and cytotoxic T-lymphocyte-associated protein (CTLA)-4. Despite substantial and excellent preclinical backing, most clinical studies have failed to prove the efficacy of antibody-based immune checkpoint blockades (reviewed in^[18,19,199-202]^). We^[146]^ and a recent series of reviews^[151,154,155]^ have detailed the reported evidence showing that GSK3β attenuates the ability of anti-tumor immunocellular arsenals such as NK cells, CD8+ T-cell-derived pluripotent memory stem T-cells with cytotoxic capacity, and tumor-type specific genetically engineered chimeric antigen receptor (CAR)-T cells. The reported evidence also showed that GSK3β enhances PD-1 expression depending on the transcription factor TBX21 (Tbet) and PD-L1 expression in response to the inhibition of poly [ADP-ribose] polymerase (PARP)1, and that inhibition of GSK3β reverses the blockade of CD28’s ability to bind and stimulate antigen-presenting immune cells by CTLA-4. Importantly, a recent study demonstrated that FAK suppresses antigen processing and presentation to promote immune evasion in pancreatic cancer^[203,204]^, suggesting a previously underexplored mechanistic link between desmoplastic tumor stroma and tumor cell-autonomous mechanisms of immune evasion^[205-207]^. If future studies provide a direct relationship between tolerant anti-tumor immunity and the acquisition of chemoresistance, it will enhance the opportunity to combat chemoresistance in pancreatic cancer by targeting GSK3β.

## CONCLUSION

GSK3β potentially functions as a molecular hub that wires chemoresistance, tumor invasion, and cancer stemness phenotype, thereby aggravating pancreatic cancer towards the incurable/devastating disease stage.

## DECLARATIONS

### Copyright

© The Author(s) 2024.
