# Supplementary material for: Glycogen synthase kinase 3β: the nexus of chemoresistance, invasive capacity, and cancer stemness in pancreatic cancer
Source: Cancer Drug Resist. 2024 Jan 31;7:4. doi: 10.20517/cdr.2023.84 (PMC10838383; doi:10.20517/cdr.2023.84)
Supplement: Supplementary file 1 [file cdr-7-4-SupplementaryMaterials.pdf]

Uehara *et al.* *Cancer Drug Resist* 2024;7:4
DOI: 10.20517/cdr.2023.84

Cancer Drug Resistance

Supplementary Materials

Glycogen synthase kinase 3β: the nexus of chemoresistance, invasive capacity, and cancer stemness in pancreatic cancer

Masahiro Uehara[1,#], Takahiro Domoto[1,#], Satoshi Takenaka[1,2,3], Osamu Takeuchi[4], Takeo Shimasaki[1,5], Tomoharu Miyashita[1,2,3], Toshinari Minamoto[1]

[1]Division of Translational and Clinical Oncology, Cancer Research Institute, Kanazawa University, Kanazawa 920-0934, Japan.
[2]Department of Hepato-Biliary-Pancreatic Surgery and Transplantation, Graduate School of Medical Sciences, Kanazawa University, Kanazawa 920-8641, Japan.
[3]Department of Surgery, Toyama City Hospital, Toyama 939-8511, Japan.
[4]Biomedical Laboratory, Department of Research, Kitasato University Kitasato Institute Hospital, Tokyo 108-8642, Japan.
[5]Medical Research Institute, Kanazawa Medical University, Uchinada 920-0293, Japan.
[#]Authors contributed equally.

Correspondence to: Dr. Toshinari Minamoto, Division of Translational and Clinical Oncology, Cancer Research Institute, Kanazawa University, 13-1 Takara-machi, Kanazawa 920-0934, Japan. E-mail: minamoto@staff.kanazawa-u.ac.jp

Supplementary Table 1. The previously reported representative mechanisms and associated factors for resistance to gemcitabine in pancreatic cancer[9-11,22-25]

Categories	Responsible factors	Mechanisms for resistance to gemcitabine	Ref.
Cellular transporters for drug uptake and efflux	hENT ↓	Restriction of uptake of gemcitabine via reduced expression of hENT1, a nucleoside transporter functioning of intracellular uptake of gemcitabine.	[26-28]
	MRP5 ↑	Enhancement in efflux of gemcitabine by MRP5, a member of ABC transporter family responsible for extracellular transport of gemcitabine*	[28,29]
Drug metabolism	dCK ↓	Inactivation of dCK, a rate-limiting kinase for metabolic activation of dFdC/gemcitabine	[30,31]
	HuR ↓	Downregulation of dCK via reduced expression of HuR, an RNA-binding protein that post-transcriptionally stabilizes dCK mRNA	[32]
	RRM1 ↑	Increased expression and activity of RRM1 (a rate-limiting enzyme for DNA synthesis) that is inhibited by dFdCDP, an intermediate metabolite of dFdC/gemcitabine	[33-35]
Anti-apoptotic pathways	PI3K/Akt ↑	PI3K/Akt-mediated pathway protects PDAC cells from apoptosis induced by gemcitabine	[36]
	BNIP3 ↓	Downregulation of BNIP3, a member of the Bcl-2 family, inactivates the apoptotic mediators, resulting in resistance	[37]
	ILK ↑	Overexpression of ILK decreases caspase 3 expression, resulting in gemcitabine resistance	[38]
	MUC4 ↑	Overexpression of MUC4 activates the MUC4-HER2-mediated anti-apoptotic pathway	[39]
	CEACAM6 ↑	CEACAM6 activates Akt/PKB in a c-Src-dependent manner, protecting PDAC cells from cytochrome-c-induced caspase 3 activation	[40]
	p8 ↑	Overexpression of p8, a stress associated and	[41]

		anti-apoptotic protein, deactivates caspase 3	
Pro-oncogenic pathways	MAPK	ERK and neuropilin-1 (a co-receptor for VEGF) confer gemcitabine resistance on PDAC cells via induction of MAPK signaling cascade<br>PI3K/Akt-mediated pro-survival pathways protect PDAC cells from apoptosis<br>Src tyrosine kinase is responsible for gemcitabine resistance via activating the Akt-mediated pathway	[36,42-45]
	NF-κB	NF-κB-mediated pro-survival pathways protect PDAC cells from apoptosis	[45-48]
	Notch	Notch-2 and its ligand, Jagged-1, are responsible for EMT phenotype in gemcitabine-resistant PDAC cells. Notch 3 induces gemcitabine resistance via activation of the PI3K/Akt-dependent pathway	[49,50]
	Hedgehog	Hedgehog signaling causes fibrosis and decreases tumor vascular density, reducing the delivery of gemcitabine	[51]
EMT	ZEB1, slug	ZEB1 and slug, the repressors of E-cadherin, induce gemcitabine resistance	[52]
	Twist	The level of twist was increased in nuclear fraction of gemcitabine-resistant PDAC cells	[53]
TME		TME components consisting of mesenchymal (e.g., PSCs, CAFs), inflammatory and immune cells embedded in desmoplastic fibrous stroma orchestrate the physically and biologically obstinate barrier against the chemotherapeutics including gemcitabine and the immunotherapeutic regimens<br>Gemcitabine resistance is induced by intratumor Gammaproteobacteria, a class of microbiome, dependent on bacterial CDD_L expression, and	Reviewed in[54-62]<br>[63]

	abrogated by cotreatment with the antibiotic ciprofloxacin

*A controversial result was previously reported in human embryonic kidney (HEK)293 cells {Adema AD, Floor K, Smid K, Honeywell RJ, Scheffer GL, Jansen G, Peters GJ. Overexpression of MRP4 (ABCC4) and MRP5 (ABCC5) confer resistance to the nucleoside analogs cytarabine and troxacitabine, but not gemcitabine. Springerplus 2014;3:732. [PMID:25674464 DOI:10.1186/2193-1801-3-732]}; ↓: decreased expression or activity; ↑: increased expression or activity. ABC: ATP binding cassette; ATP: adenosine triphosphate; Bcl2: B-cell/CLL lymphoma 2; BNIP3: Bcl2/adenovirus E1B 19 kDa protein-interacting protein 3; CAF(s): cancer-associated fibroblast(s); CDD: cytidine deaminase; CDD_L: a long isoform of bacterial CDD; CEACAM6: carcinoembryonic antigen cell adhesion molecule 6; CLL: chronic lymphocytic leukemia; dCK: deoxycytidine kinase; dFdC: 2',2'-difluoro-2'-deoxycytidine; dFdCDP: dFdC diphosphate; ECM: extracellular matrix; EMT: epithelial-mesenchymal transition; ERK: extracellular signal-regulated kinase; hENT: human equilibrative nucleoside transporter; HER2: human epidermal growth factor receptor 2; HuR: Hu antigen R; ILK: integrin-linked kinase; MAPK: mitogen-activated protein kinase; MRP5: multidrug resistant protein 5; NF-κB: nuclear factor-κB; PI3K: phosphatidylinositide 3-kinase; PSC(s): pancreatic stellate cell(s); RRM1: ribonucleotide reductase subunit M1; TME: tumor microenvironment; VEGF: vascular endothelial growth factor; ZEB1: zinc-finger-enhancer binding protein 1.
